# Rastafarianism: When Religious Beliefs Conflict With Medical Necessity—A Case Report and Review of the Literature Around an Ethically Complicated Case

**DOI:** 10.1155/crps/6621450

**Published:** 2025-03-18

**Authors:** Matthew Flick, Casey Martinez, David C. Fipps

**Affiliations:** ^1^Mayo Clinic Alix School of Medicine, Mayo Clinic Arizona, Scottsdale, Arizona, USA; ^2^Department of Psychiatry and Psychology, Mayo Clinic, Rochester, Minnesota, USA

## Abstract

**Background:** Rastafarianism maintains that cannabis is a sacred element of the religious practice, and followers of the religion traditionally engage cautiously with western medicine. This case involves Mr. I, a 72-year-old Rastafari male with acute myelogenous leukemia (AML) and hepatic decompensation, who developed delirium with psychotic features which were initially managed with quetiapine. His family expressed concerns with psychotropic medications and requested using dronabinol, a synthetic cannabinoid, to manage his symptoms considering the spiritual significance of cannabinoids in the Rastafari culture. The psychiatry team's dissenting recommendations regarding dronabinol was met with resistance, and the family voiced that they felt their religious beliefs were not being respected and considered bringing in their own marijuana products. Following an ethics consultation, a compromise was reached to trial low-dose dronabinol. However, Mr. I's symptoms worsened, prompting discontinuation of dronabinol and management with olanzapine.

**Discussion:** This case exemplifies the complexities of clinical care when religious beliefs conflict with medical necessity. We discuss the limited indications for dronabinol and potential adverse effects on delirium's behavioral symptoms. Concerns about fungal sensitization from cannabinoid products in the context of immunosuppressive chemotherapy and the effects of cannabinoids on hepatic dysfunction are also explored. Moreover, we emphasize the importance of cultural sensitivity for Rastafari individuals who view marijuana as sacred and therapeutic. Balancing cultural and religious sensitivity with ethical, evidence-based medicine through a thorough discussion of risks and benefits is essential for optimal decision-making in such ethical dilemmas.

## 1. Introduction

Rastafarianism is a religious and cultural movement with its origin in the 1930s Jamaica which holds significance for Black individuals, emphasizing affirmation of African identity. Ras Tafari Makonnen—later, Emperor Haile Selassie I of Ethiopia—is often honored as a divine figure within the culture, hence influencing the name of the movement. Individuals who practice Rastafarianism follow a diverse set of beliefs and practices, with many viewing western medicine as overly reliant on pharmaceuticals and invasive treatments. Additionally, a central aspect of the religion is the use of herbal remedies (predominantly marijuana) as a sacrament and a tool for healing, spiritual awakening and purification, consciousness raising, and worship. Often referred to as “ganja,” marijuana refers to the dried constituents of the cannabis plant, which is a genus of flowering plant that contains compounds such as the psychoactive tetrahydrocannabinol (THC) and nonpsychoactive cannabidiol (CBD). Cannabis products have been used for multiple complaints through recreational formulations such as marijuana, cannabis gummies, CBD oil, lotions, etc., and through pharmacologic formulations. To date, the U.S. Food and Drug Administration (FDA) has approved CBD formulations (Epidiolex) for treatment of seizures associated with Lennox–Gastaut syndrome, nabilone formulations (Cesamet) for treatment of nausea and vomiting associated with cancer chemotherapy, and dronabinol formulations (Marinol and Syndros) for anorexia and weight loss in the setting of known diagnosis of human immunodeficiency virus (HIV) and acquired immunodeficiency syndrome (AIDS) as well as chemotherapy-induced nausea/vomiting [1, 2]. The evidence for efficacy of FDA-approved formulations of cannabis in the context of critical medical illness is limited. Furthermore, a recent large systematic review and meta-analysis called attention to the great variability in literature-supported evidence, therapeutic benefit, and adverse effects of recreational cannabis use [3]. Finally, there is a dearth of peer-reviewed medical literature on Rastafarianism and how the beliefs intertwine with complex medical decision making.

Here, we present a uniquely significant case that exemplifies the ethical and clinical dilemma where attempting to respect Rastafari beliefs conflicted with medical necessity. This review aims to provide a risks and benefits analysis and examine relevant literature surrounding care of individuals with beliefs that conflict with conventional western medicinal practices and contributes a knowledge base for complex decision-making regarding care for critically ill Rastafari individuals who use cannabinoids. While there have been studies addressing similar dilemmas in patients of other faiths, this review is likely the first and most comprehensive to explore the considerations of cannabinoid use by Rastafari individuals in a western medical setting and aims to fill a gap in culturally sensitive medical literature which has previously been overlooked.

## 2. Case Description

Mr. I was a 72-year-old married male with no previous psychiatric diagnoses, suicide attempts, or psychiatric hospitalizations who was recently diagnosed with acute myelogenous leukemia (AML). He had a history consistent with strong religious beliefs that led to no major interaction with western medicine throughout his life, instead following alternative medicinal practices. He presented through an interfacility transfer with a 1-week history of right flank pain, fever, and shortness of breath. Initial workup at the outside facility revealed leukocytosis with blasts of 30% and a nodular liver consistent with cirrhosis. Further investigations, including a bone marrow biopsy, confirmed a diagnosis of AML with poor prognostic factors. Additionally, an in-house liver biopsy confirmed AML infiltration with sinusoidal involvement.

Upon admission to the hematology–oncology service, Mr. I began exhibiting symptoms of delirium, including waxing and waning confusion, orientation, attention deficits, tactile hallucinations (bugs crawling on skin), agitation, and restlessness, in addition to loss of appetite and anxiety with elevated heart rate. The primary team initiated treatment with 25 mg p.o. four times a day of quetiapine to manage these symptoms with as-needed dosing for acute agitation. However, after having received two doses, Mr. I's family expressed concerns about using psychotropic medications and preferred alternatives to western medicine to address his symptoms. With these concerns, Mr. I's wife and daughter raised the possibility of using dronabinol, as they felt it would be the best option to address his symptoms and potentially improve his overall comfort level. They emphasized that Mr. I was a devout Rastafarian and had been using marijuana and cannabis gummies as part of his cultural and spiritual practices throughout his life. Consequently, the primary team sought a psychiatric consultation to assess Mr. I's mental status and the appropriateness of using dronabinol in this context.

Consistent with the waxing and waning nature of delirium, when the psychiatry consult service evaluated the patient, his attention and awareness testing were intact, and he was alert and oriented to person, time, place, and situation. In this consult, he voiced a willingness to undergo chemotherapy for his recent cancer diagnosis, despite his discomfort with western medicine practices. Additionally, Mr. I did not report feelings of depression, anxiety, or suicidal thoughts. He noted that his sleep and confusion had greatly improved during the timeframe of scheduled quetiapine; however, his insight into this connection was quite poor. In fact, his family reiterated their concerns about psychotropic medications and felt that the correlation of tactile hallucinations peripheral to quetiapine administration was just cause to support its discontinuation. They maintained their desire to start dronabinol and cited that cannabis held a central and sacred place in Rastafari culture and they felt that their religious beliefs were not being respected by delaying such treatment. In fact, Mr. I's family vehemently iterated that they planned to bring in their own home-grown cannabis formulations if their requests were not fulfilled.

Ultimately, the psychiatry team gave three recommendations: (1) They held that the risks associated with using dronabinol in the context of his delirium and medical comorbidities were extremely high and that THC/cannabinoid preparations may further contribute to or exacerbate his delirium symptoms. (2) It was initially suggested to replace quetiapine with olanzapine as a compromise; however, Mr. I's family was not interested in this medication change. (3) Consult ethics regarding the conflict of Mr. I's religious beliefs and medical recommendations.

The ethics consultation acknowledged the need to respect cultural and religious beliefs while ensuring the delivery of appropriate medical care. Discussions centered on the need to treat Mr. I's AML aggressively, benefiting greatly from his stay in the hospital. Additionally, the family's request for Marinol specifically to reduce his symptoms of delirium and tachycardia was met with uncertainty, as the ethics team cited Marinol's primary use for treating nausea and vomiting associated with chemotherapy. They identified that healthcare providers are not obligated to prescribe treatments they consider nonbeneficial or potentially harmful. In a subsequent multidisciplinary care conference, the team expressed multiple concerns regarding marijuana and its influence on outcomes with patients who have compromised immune systems. Additionally, concerns were raised about the inconsistent dosing of home-grown cannabis as well as the questionable efficacy of dronabinol in managing delirium symptoms. Additional points were raised regarding the terminal nature of Mr. I's condition, which created a barrier to hospital transfer. Considering the family's continued insistence on respect for their culture and religion, and safety concerns if they were to bring in home-grown marijuana, the team decided to propose a compromise. It was decided to start a time-limited low dose (2.5 mg nightly) of dronabinol with clear parameters to track regarding desired efficacy or worsening of symptoms (e.g., heart rate, anxiety, agitation, appetite, etc.).

Only days after starting dronabinol, Mr. I experienced a notable spike in his liver function tests as well as fluctuating bilirubin levels. This prompted a transplant hepatology consultation to assess his eligibility for a liver transplant. The hepatology team identified multiple potential etiologies for his transaminitis with suspicion of drug-induced liver injury versus leukemia infiltration vs. combination. A liver biopsy showed findings consistent with infiltration by his known AML, and he was not considered an appropriate transplant candidate due to likely metastatic disease. Furthermore, Mr. I's heart rate variability, anxiety, and agitation did not improve with dronabinol, in fact, these symptoms worsened, even in tandem with slight dosing optimization (adding an additional 5 mg of dronabinol once every morning). Considering this, Mr. I's wife agreed to add 2.5 mg of olanzapine to his regimen and an eventual wean off dronabinol. This led to a substantial clinical improvement in his delirium and anxiety symptoms, with near complete resolution after the cross-titration was finalized. The remainder of his hospital course was pertinent for intermittent mild delirium symptoms that gradually continued to improve as he progressed through his course of chemotherapy (consisting of decitabine and venetoclax). With his overall clinical picture improved and an updated bone marrow biopsy confirming no further evidence of acute leukemia, he was discharged to an outpatient level of care.

## 3. Discussion

This case exemplifies the complexities of clinical care when religious beliefs conflict with medical necessity. We use this case as an example of complex decision making for this manuscript, and it is not necessarily meant to be a guide for best practices. This discussion will explore the limited indications for dronabinol, alongside addressing concerns about its potential impact on the behavioral symptoms of delirium and psychosis risk in medically complicated patients. We further discuss how the increased risk of fungal infection associated with use of cannabinoid products may particularly threaten patients undergoing immunosuppressive chemotherapy. Additionally, we explore the proposed role of cannabinoids in hepatic dysfunction. Finally, we will delve into the ethical concern surrounding cultural sensitivity for Rastafari individuals, who view marijuana as culturally sacred and therapeutic.

### 3.1. CB Receptor Activity

There are two pharmacologically pertinent cannabinoid receptors named CB1 and CB2. CB1 is located primarily in the central and peripheral nervous systems (CNS, PNS) and parts of the gastrointestinal system, while the CB2 receptor is primarily located in cells of immunologic origin. Dronabinol agonizes the CB1 receptor, which directly inhibits G-protein coupled receptors imparting the psychoactive effects of cannabis [1, 4]. CB1 receptors are known to inhibit the release of excitatory neurotransmitters in addition to localizing presynaptically on gamma-aminobutyric acid (GABA) interneurons, where they inhibit neurotransmitter release in both the CNS and PNS [5]. The activation of CB1 receptors is thought to mediate the subjective calming and anxiolytic effects associated with cannabis consumption [6]. However, chronic cannabinoid exposure has been shown to lead to tolerance through downregulation of CB1 receptors within neurons of the nucleus accumbens, reducing sensitivity of the endocannabinoid system and decreasing levels of endocannabinoids normally involved in regulating mood, appetite, and stress response. As a result, withdrawal from cannabinoids can lead to worsened sleep, anxiety, and appetite. In Mr. I's case, the use of dronabinol to address anxiety and agitation, despite its CB1 agonism, appeared to exacerbate his symptoms, potentially reflecting the effects of chronic cannabinoid exposure and endocannabinoid receptor desensitization.

### 3.2. Appetite and Emesis

As seen in this case, a goal of dronabinol was to improve the patient's appetite. CB1 receptors in the hypothalamic nuclei, particularly the arcuate nucleus, stimulate appetite and enhance the rewarding aspects of food [7]. Additionally, both THC and CBD modulate CB1 receptors in the area postrema of the brainstem, a center involved in triggering emesis in response to noxious stimuli. However, a reciprocal effect exists where contrary to its antiemetic property at lower doses, the consumption of high doses of cannabis has paradoxically been linked to episodic nausea and vomiting, termed cannabis hyperemesis syndrome, which may lead to severe dehydration and in some cases Wernicke's encephalopathy [8]. While dronabinol was intended to stimulate Mr. I's appetite, his lack of response and worsening agitation brought concern about the limited utility of cannabinoids in managing such symptoms in a medically fragile patient.

### 3.3. Drug Interactions

Pharmacologically, THC and CBD act as inducers of cytochrome P450 (CYP) 1A2 and inhibitors of CYP3A4, 3A5, 2D6, and P-glycoprotein (P-GP). These interactions can bring clinical pertinence, particularly by influencing blood levels of medications and substances processed through these systems. In our case, the patient was taking venetoclax, a known CYP3A4 substrate, thus any cannabinoid product would hold the potential for metabolic interactions. Furthermore, venetoclax also inhibits P-GP, which would cause a synergistic interference with substrate absorption [9].

### 3.4. Therapeutic Potential and Risks

As noted above, there is great variability in literature-supported evidence of both therapeutic benefits and adverse effects based on the type of cannabinoid utilized [3]. Additionally, the risk-analysis for cannabis may vary unfavorably for treating certain conditions, as one meta-analysis regarding cannabis consumption for chronic pain management reported a number needed to treat 24 with a number needed to harm of only six [10]. Moreover, it is key to note that all clinical studies demonstrating evidence for the therapeutic administration of cannabinoids used synthetic pharmaceutical-grade cannabis, not smoked marijuana, and much is left to be investigated regarding whether the medicinal benefits extend to nonpharmaceutical grade cannabis [11]. Furthermore, according to the American Psychiatric Association, “There is no current scientific evidence that cannabis is in any way beneficial for the treatment of any psychiatric disorder” [12]. There is clear evidence of the risk of addiction to cannabinoid products; the Marinol package insert notes “ingestion of high doses of dronabinol increases the risk of psychiatric adverse reactions if abused or misused, while continued administration can lead to addiction” [13]. In Mr. I's case, pharmaceutical grade dronabinol, which in theory reduced several risks associated with alternative cannabinoid formulations, was still associated with worsening of symptoms and no therapeutic benefit, further depicting the difficulty in assessing potential benefits of such a therapeutic in complex psychiatric and medical conditions.

### 3.5. Contaminants and Labeling

Even medical-grade marijuana use comes with risks as there is no governmental oversight for quality or purity, and this is even more pertinent with homegrown marijuana as proposed by the family in our case [14, 15]. In an analysis of 84 medical marijuana products, Vandrey et al. [16] found contaminants in 21% of THC extracts, all of which contained compounds not explicitly noted in the product label. In addition, only 17% of the labels were accurate in their CBD and THC concentrations, where 23% over-reported the label's concentration values and 60% under-reported [16]. A 2024 report of retail cannabis products in Illinois found that 93% of tested products had inaccurate labels, including some with no cannabinoids at all. Further, some labels were found to have 456% more THC than what was listed on the package (some containing up to 7000 mg of THC, when there is a regulated limit of 100 mg per packet in Illinois) [17]. At the time of writing this report, contaminants and inaccurate labeling of nonpharmaceutical cannabis products appear to be the rule rather than the exception, thus further increasing the risk if used in medically complex situations [18].

### 3.6. Patients With Immunocompromise

As synthetic cannabinoids are clinically approved for patients with HIV/AIDS and during chemotherapy, their use is common in individuals with immunocompromise. The use of cannabis products significantly increases infection risk through two main avenues: fungal spores and pesticide residues [19]. Smoking marijuana bears the most risk of infection; however, sensitization risks are still evident with vaping and edibles [20]. Furthermore, the sterilization processes employed in medical marijuana are not always effective in neutralizing fungal spores [19]. One study analyzing medical marijuana samples from various Californian dispensaries found several viable species of Gram-negative bacteria and fungal pathogens, including cryptococcus and aspergillus [18]. Though rare, marijuana may cause potentially devastating infections in patients with weakened immune systems, with one case report of a bone marrow transplant recipient dying due to fungal infection contracted from smoking marijuana [21]. These findings are even notable in patients without immunocompromise. Though understudied, the immunosuppressive role of CB2 receptors on microglial cells within the CNS could contribute to the underlying pathophysiology of fungal sensitization and vulnerability [22]. As seen in this case, the patient was in an immunocompromised state, thus increasing the risk of potential morbidity and mortality from such attributes of cannabis products, especially from home-grown marijuana. Though risk stratification amongst cannabinoid preparations has not been clearly elucidated in the literature, using a prescription medication, such as dronabinol, could theoretically lower the risk of fungal sensitization compared to nonregulated cannabinoid products (especially in high-risk patients who have immunocompromise).

### 3.7. Cannabinoids and Liver Injury

Drug induced liver injury (DILI) initially emerged as a critical concern in the case of Mr. I, where the concomitant initiation and dose increase of dronabinol were correlated in time with peaking liver function tests and fluctuating bilirubin levels, raising suspicion that Mr. I's impaired liver function could have been due to factors beyond AML infiltration. Current evidence regarding liver health suggests that hepatocyte and endothelial cell CB1 receptors have a role in promoting hepatic fibrogenesis, proinflammatory states, and hepatic steatosis, raising concern over the synergistic and potentially damaging effects of cannabinoid use with pre-existing hepatic injury [23–25]. However, the overall clinical influence of cannabinoids on liver health is not fully clear, as CB2 receptors have been shown to have anti-inflammatory properties which theoretically could have a beneficial effect on hepatic inflammation and fibrosis [22]. Further clinical studies are needed to accurately assess the influence of cannabinoids on overall hepatic risks.

### 3.8. Delirium and Psychosis

Cannabinoids have been linked to increased adverse psychiatric reactions such as anxiety, insomnia, depression, suicidal behavior, hallucinations, and mood fluctuations [26]. Dronabinol's own package insert further underscores these concerns, highlighting the potential for additional psychiatric symptoms such as depersonalization, paranoia, and physical dependence from chronic therapy [13]. Moreover, the endocannabinoid system's involvement in hepatic encephalopathy complicates the assessment and management of delirium in patients with hepatic dysfunction [27–29]. Beyond this, there is no current data on dronabinol's efficacy in managing the behavioral sequelae of delirium [12]. Even a single dose of cannabis has shown an ability to induce psychosis [1, 13]. Outside of acute intoxication, daily cannabinoid use has been linked to a 3.2-fold (95% CI = 2.2, 4.1) increased risk of psychosis while high-potency cannabinoid products show an odds ratio of 4.8 (95% CI = 2.5, 6.3) [30–33]. Cannabis consumption has been independently linked to an increased risk for the development of primary psychotic disorders, with a higher conversion rate to schizophrenia than for individuals using any other substance and a 163-fold increased risk relative to the general population [34]. Antipsychotics, such as haloperidol, olanzapine, and quetiapine, are commonly used when managing the behavioral sequalae of delirium, particularly when agitation, hallucinations, and/or delusions dominate the clinical picture [35, 36]. As seen in our case, the initiation of dronabinol was correlated to a clear worsening of psychotic symptoms and mood dysregulation, and these symptoms greatly improved with its subsequent discontinuation and implementation of a more evidence-based antipsychotic agent.

### 3.9. Cannabis Use Disorder and Cannabis Withdrawal

When assessing cases where cannabis or cannabis-based treatments are used regularly, especially as part of a cultural practice, assessing for the possibility of a substance use disorder (SUD) and withdrawal is critical. This information provides a holistic view of potential outcomes for the patient and aids ethical decision-making. The Diagnostic and Statistical Manual of Mental Disorders, Fifth Edition, Text Revision (DSM-5-TR) outlines criteria for diagnosing SUD, including tolerance, withdrawal symptoms, unsuccessful efforts to cut down, and continued use despite psychological or physical harm [37]. Due to the acuity of Mr. I's medical conditions and active delirium symptoms, the formal assessment of Mr. I's potential for cannabis use disorder was quite limited. If a SUD is suspected in similar ethical situations, this further accentuates the importance of identifying the risk of continued use of addicting substances and would likely sway the final risk and benefit conversation toward avoidance of the proposed agent.

Cannabis withdrawal symptoms can include irritability, sleep disturbance, reduced appetite, and restlessness. Patients who regularly use cannabis may experience withdrawal if their usage is stopped or reduced abruptly. As seen in this case, Mr. I exhibited symptoms that could be consistent with cannabis withdrawal (agitation, restlessness, loss of appetite, anxiety, and poor sleep); however, there were multiple additional variables that could have contributed to this clinical picture. Though there is evidence of successful use of dronabinol to address cannabis withdrawal symptoms, the overall complexity of Mr. I's case warranted consideration of other contributing factors and safer alternatives; agents such as gabapentin and buspirone have been used off-label to address cannabis withdrawal symptoms and could have been appropriate first line considerations in Mr. I's case [38–40].

### 3.10. Ethical Conflict: Rastafarianism and Dronabinol

This case and subsequent ethical dilemma brings forth an appropriate consideration of the ethical principles of autonomy, beneficence, justice, and nonmaleficence. In addition, when faced with clinical decisions where the next best step may be in conflict with a patient's religious beliefs, a thorough risk, benefit, and alternatives discussion with informed consent is paramount. To reiterate, our case is not meant to represent best practices, but rather to act as an example to springboard a thorough risk/benefit analysis where boundary setting may be appropriate.

To begin, the benefits of dronabinol use in this case represented an attempt to best respect the patient's autonomy as well as cultural and religious beliefs of the patient and family members. Additionally, the long-term success of any doctor–patient relationship requires respect and consideration of appropriate shared decision-making strategies. However, in this case, the patient's fluctuating delirium hindered his decision-making capacity, shifting weight onto the requests expressed by his family, and complicating the patient's ability to make an autonomous decision.

Physicians must also incorporate beneficence when rectifying patient preferences with medical necessity. Dronabinol is a reasonable choice to stimulate appetite. Furthermore, the use of dronabinol could have potentially mitigated any underlying cannabis withdrawal, which could have contributed to his discomfort, agitation, and delirium severity. Throughout the early stages of his hospital stay, the patient's family saw dronabinol's implementation as potentially beneficial for his wellness. Acting in the patient's best interest for their continued hospital stay and to minimize potential risks associated with the use of homegrown cannabis, the team implemented a trial of dronabinol with strict monitoring for possible adverse effects to avoid undue harm while acknowledging its potential benefits for Mr. I.

Furthermore, justice involves ensuring fair and impartial treatment for each patient, which often requires balancing a patient's cultural needs and medical guidelines. In this case, the team selected a treatment plan that they felt best ensured an equitable distribution of standard-of-care treatment to Mr. I for his AML throughout an in-patient timeline, which relied upon the family's perception of the care team's cultural competence regarding Rastafari practices. This pivotal conflict motivated the team to provide comprehensive treatment for the acute and long-term well-being of the patient.

Finally, as was noted by the ethics team in the case description, physicians should not feel pressured to prescribe medications they deem potentially harmful to patients—as noted in the Hippocratic oath, first “do no harm” (nonmaleficence) [41]. There were many risks and vulnerabilities pertinent to this case that brought concerns about using a cannabis/THC product. First, there could be potential interactions with Mr. I's chemotherapy regimen. Second, the risk of fungal sensitization could be more dangerous due to his compromised immune system. Third, the safety profile of cannabis/THC products in hepatic impairment is limited and potentially dangerous. Lastly, his active psychotic symptoms from delirium were vulnerable to exacerbation with any potentially psychoactive agent. As seen in the case, dronabinol was correlated to clinical worsening of his delirium, and these symptoms improved with its discontinuation.

### 3.11. Similar Medical Conflicts With Patient Culture

In many cultures, religious and spiritual beliefs can conflict with medical practice, creating complicated ethical dilemmas for healthcare providers. Highlighting such a conflict, this case describes a Rastafari patient requesting dronabinol, though similar situations regarding other cultural contexts have been described in medical ethics literature. For instance, multiple African communities practice their own traditional forms of medicine, including herbal treatments, rituals, and spiritual healing, akin to Rastafarianism [42]. Similar to this case, patients may prefer traditional remedies alongside or instead of western medicine, leading to potential negative interactions with concurrent treatments or the patient's medical condition.

As an ethical counterpart to requests for additional, culturally aligned, treatment, patients may also refuse or object to recommended treatment on the basis of their faith. For instance, in Islam, a fundamental religious practice is fasting during Ramadan [43, 44]. For patients with diabetes who practice Islam, fasting may pose serious health risks, such as hypoglycemia and hyperglycemia, and healthcare professionals must balance respect for such patients' religion with a responsibility to prevent harm in this population. Similarly, followers of orthodox Judaism may avoid electronic devices while on the Sabbath [45]. During this time, prohibition against using medical devices for patients requiring critical treatment or monitoring warrants careful negotiation between observation of the patient's religious duties and recommendation for medical care. Regarding the broader practice of medicine, Jainism acknowledges a religious commitment to nonviolence and nonharm to animals which may conflict with the production of conventional medicines such as vaccines or other life-saving treatments. However, followers of Jainism have developed tools for carefulness and nonviolence in receiving medicine that accommodates both their religious beliefs and medical necessity [46]. As each conflict of this nature requires unique consideration for a patient's background, these challenges should be increasingly studied and anticipated by healthcare professionals to best ensure appropriate and safe medical treatment is provided.

### 3.12. Future Studies

Future studies could elaborate upon the shared decision-making process regarding cannabinoid use with Rastafari individuals, how outcomes for these individuals vary in the hospital setting (with and without continuous use of cannabinoid products), and could explore the risks of cannabinoid use in the context of hospital delirium irrespective of the cultural and religious perspectives.

## Data Availability

Data sharing is not applicable as no new data were generated.
